# Causal Association between Periodontitis and Parkinson’s Disease: A Bidirectional Mendelian Randomization Study

**DOI:** 10.3390/genes12050772

**Published:** 2021-05-19

**Authors:** João Botelho, Vanessa Machado, José João Mendes, Paulo Mascarenhas

**Affiliations:** 1 Centro de Investigação Interdisciplinar Egas Moniz (CiiEM), Periodontology Department, Clinical Research Unit (CRU), Egas Moniz—Cooperativa de Ensino Superior, CRL, 2829-511 Almada, Portugal; vmachado@egasmoniz.edu.pt; 2 Evidence-Based Hub, CRU, CiiEM, Egas Moniz—Cooperativa de Ensino Superior, CRL, 2829-511 Almada, Portugal; jmendes@egasmoniz.edu.pt (J.J.M.); pmascarenhas@egasmoniz.edu.pt (P.M.); 3 Center for Medical Genetics and Pediatric Nutrition Egas Moniz, IUEM, 2829-511 Almada, Portugal

**Keywords:** Parkinson’s disease, periodontitis, periodontal disease, Mendelian randomization, bioinformatics, oral health

## Abstract

The latest evidence revealed a possible association between periodontitis and Parkinson’s disease (PD). We explored the causal relationship of this bidirectional association through two-sample Mendelian randomization (MR) in European ancestry populations. To this end, we used openly accessible data of genome-wide association studies (GWAS) on periodontitis and PD. As instrumental variables for periodontitis, seventeen single-nucleotide polymorphisms (SNPs) from a GWAS of periodontitis (1817 periodontitis cases vs. 2215 controls) and eight non-overlapping SNPs of periodontitis from an additional GWAS for validation purposes. Instrumental variables to explore for the reverse causation included forty-five SNPs from a GWAS of PD (20,184 cases and 397,324 controls). Multiple approaches of MR were carried-out. There was no evidence of genetic liability of periodontitis being associated with a higher risk of PD (B = −0.0003, Standard Error [SE] 0.0003, *p* = 0.26). The eight independent SNPs (B = −0.0000, SE 0.0001, *p* = 0.99) validated this outcome. We also found no association of genetically primed PD towards periodontitis (B = −0.0001, SE 0.0001, *p* = 0.19). These MR study findings do not support a bidirectional causal genetic liability between periodontitis and PD. Further GWAS studies are needed to confirm the consistency of these results.

## 1. Introduction

Parkinson’s disease (PD) is a neurodegenerative condition with heterogeneous clinical patterns and progression [1,2]. Despite the cause of PD still not being fully understood, a comprehensive mendelian randomization (MR) study described the relative risk of developing PD for 12 different exposures [3]. Furthermore, the role of inflammation in PD, either by local or systemic causes, has been consistently studied [4].

A newly examined inflammatory cause associated with PD was periodontitis [5]. Periodontitis is a chronic inflammatory disease of the gums with worldwide impact [6]. This condition represents the local manifestation of a developing immuno-physiological disruption, results in alveolar bone loss, and can cause tooth loss [7]. The evidence for an association between PD and periodontitis is still scarce, though the two latest studies have reported that the progression of PD (through motor and non-motor deterioration) may frustrate oral hygiene and oral health [8,9]. Moreover, individuals with PD have a high risk of developing periodontitis [10,11,12,13,14], and, if periodontitis is developed, this may lead to systemic leukocytosis [5]; however, its consequences are still unknown.

The causal relation concerning periodontitis and PD risk is limited in terms of evidence, with only observational studies available. To overcome the limitations of observational trials, including the lack of randomization, Mendelian randomization (MR) might be considered via summary data from genome-wide association studies (GWAS) for causality assessment in putative exposure–outcome pathways.

MR makes use of genetic variants in the form of instrumental variables (IVs) for the exposure of interest [15]. These IVs might be exploited to reckon the exposures’ causal effect if they meet three main assumptions: (1) strongly linked with the exposure; (2) independency from the confounding factors of the observed relationship; and (3) only the exposure permits association with the outcome (absence of horizontal pleiotropy) [15,16,17]. In addition, GWAS are increasing, fostering opportunities for MR approaches. For these reasons, this study investigated whether periodontitis and PD have a causal association in European ancestry populations using a MR approach.

## 2. Materials and Methods

### 2.1. MR of Periodontitis on Risk of PD

Periodontitis data were obtained from two meta-analyses of GWAS of periodontitis. The first was sourced from a Teumer et al. [18] review of 4032 individuals (1817 periodontitis cases vs. 2215 controls) of European ancestry. Seventeen single-nucleotide polymorphisms (SNPs) were proposed as being strongly related with periodontitis based on a significant *p*-value (<5 × 10^−6^ threshold) and were used as IVs. The second, from Munz et al. [19], served as a validation set, where eight SNPs (non-overlapping with the aforementioned seventeen SNPs) were deemed to be linked with periodontitis in 12,225 individuals with European ancestry (4924 periodontitis cases vs. 7301 controls). For both studies, summary statistics are available in Supplementary File S1.

The study by Shungin et al. [20], despite being the largest GWAS study on periodontitis, was deemed unsuitable because it is a compilation of data including the UK Biobank, therefore failing the principle of independency from the confounding factors.

If linkage disequilibrium was observed among the IVs, then they were pruned using clumping R^2^ (with a 0.001 cut-off) and the IVs expressing the lowest *p*-value were retained.

Outcome summary statistics of PD were derived from one newest GWAS study by Nalls et al. (37,688 cases and 1.4 million controls) [21], in which the magnitude of effects for each IV was obtained. The study by Nalls et al. [21] is, to this point, one of the largest GWAS of PD with European ancestry and of open access using data from the UK Biobank.

### 2.2. MR of PD on Risk of Periodontitis

PD data were derived from one GWAS by Chang et al. [22] (20,184 cases and 397,324 controls) with respective summary statistics (Supplementary File S2). Forty-five SNPs were linked towards the risk of PD with a significance of <5 × 10^−8^. Statistics for all IVs were accessible in the genome studies of periodontitis, and were integrated in this analysis. The outcome summary statistics of periodontitis were sourced from the Offenbacher et al. [23] GWAS study (975 European American adult participants).

### 2.3. MR Analysis

Our analyses were carried out using R (version 3.6.1), through MRPRESSO (1.0) and TwoSampleMR (0.4.25) packages.

The presence of horizontal pleiotropic outliers was inspected using MR-PRESSO R package (pleiotropy residual sum and outlier) [24]. Next, we computed Pseudo R^2^ (proportion of variance of exposure liability explained by SNPs) and F-statistic to estimate the strength of the instruments, whenever effect allele frequency (EAF) values were present.

TwoSampleMR was run for selected SNPs’ individual lookup requests against multiple target GWAS, harmonization of effect allele across studies, linkage disequilibrium (LD) pruning, and sensitivity analyses. The option to use adequate proxy SNPs to replace exposure SNPs absent from the outcome dataset was enabled.

The causality in both conditions was tested throughout the following MR effect estimation methods: inverse-variance weighted (IVW) method (random effects) [16], weighted median [17], and MR-Egger [25], with the latter two being considered relatively robust to horizontal pleiotropy. Furthermore, we also estimated the causal effect through the MR-RAPS (robust adjusted profile score) method, due to its robustness towards weak instrument bias [26]. Horizontal pleiotropy was examined by computing the MR-Egger regression line (intercept and 95% confidence interval [CI]) [25].

The Cochran’s Q verified the heterogeneity value among the causal estimates of each SNPs for both the IVW and MR-Egger approaches. To determine if the effect was not being caused by a particular SNP in a disproportional way, we performed leave-one-out meta-analyses. MR estimates are reported as coefficient (B) and standard error (SE). Forest plots are reported as B and the 95% confidence interval. The computation of MR estimates has previously demonstrated to be proficient in the detection of a causal outcome rather than the extent of the causative effect [15].

All original clinical studies had ethical clearance from the respective Institutional Review Boards [18,19,22].

## 3. Results

### Mendelian Randomization

In this bidirectional MR approach, no outliers were detected using MR-PRESSO (*p* = 0.80, *p* = 0.39 and *p* = 0.36 for Teumer et al. [18], Munz et al. [19], and Chang et al. [22], respectively).

Using the seventeen independent SNPs for periodontitis from Teumer et al., pseudo R^2^ value was 0.119 and the average F-statistic was 21.47. Regarding the additional eight SNPs from Munz et al. [19], pseudo R^2^ and F-statistic were 0.45 and 1245.97, respectively. From the 45 SNPs as instruments for PD, some SNPs did not have EAF values, making the computation only for those who had (37) possible; therefore, pseudo R^2^ and F-statistic values were at least 0.03 and 338.44, respectively. Overall, F-statistic results suggest that the selected SNPs were adequate in strength as IVs for both periodontitis and PD.

**Figure 1:**
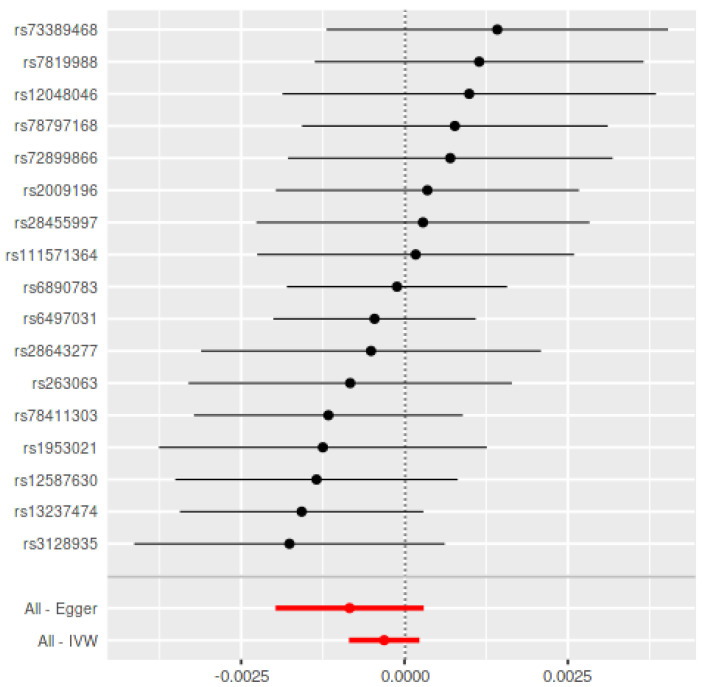
Mendelian Randomization summary effects for the risk of periodontitis associated with PD through the random-effects approach for Teumer et al. Summary effects were computed using an inverse-variance weighted (IVW) method from each individual SNP. Dots represent the coefficient and the extremities represent the 95% confidence interval of the odds ratio.

**Figure 2:**
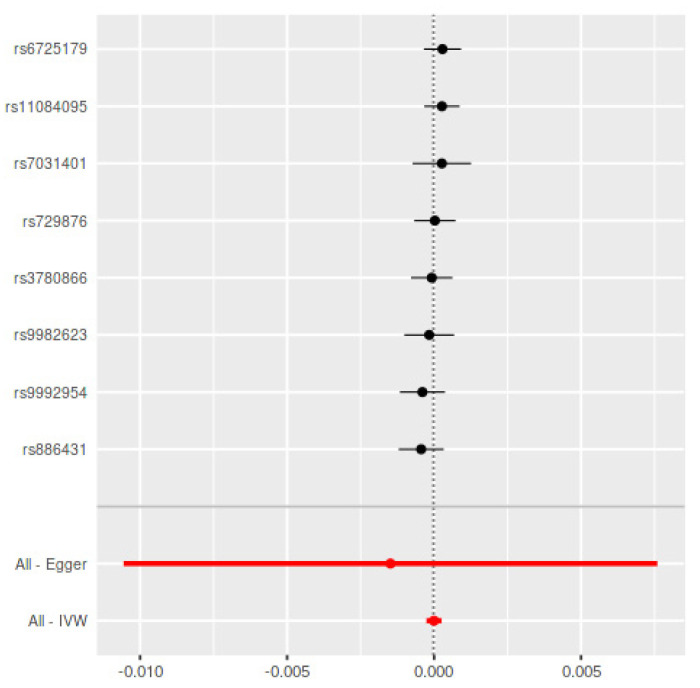
Mendelian Randomization summary effects for the risk of periodontitis associated with PD through random-effects approach for Munz et al. Summary effects were computed using an inverse-variance weighted (IVW) method from each individual SNP. Dots represent the coefficient and the extremities represent the 95% confidence interval of the odds ratio.

The bidirectional MR estimates are displayed in Table 1. Using the seventeen SNPs in Teumer et al. as periodontitis IVs, there was no association between genetically predicted periodontitis and the development of PD (B = −0.0003, SE = 0.0003, *p* = 0.26) based on the IVW estimate, which was supported by the MR-RAPS method (B = −0.0003, SE = 0.0003, *p* = 0.27) (Figure 1). In addition, the MR assessment using the weighted median (B = −0.0003, SE = 0.0003, *p* = 0.37) and MR-Egger estimate (B = −0.008, SE = 0.0006, *p* = 0.17) reported equal outcomes. The validation using eight non-overlapping SNPs in Munz et al. [19] as periodontitis IVs confirmed the aforementioned results (Table 1) (Figure 2).

**Figure 3:**
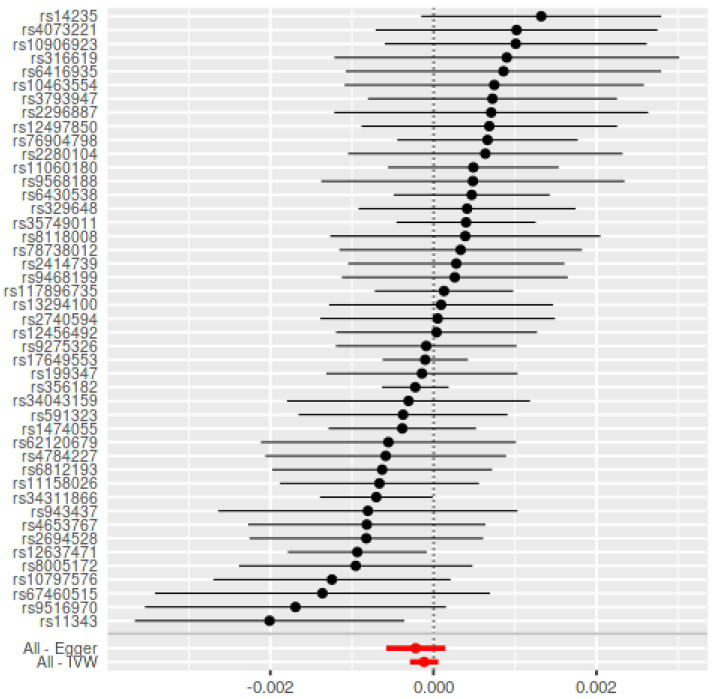
Mendelian Randomization summary effects for the risk of PD associated with periodontitis through random-effects approach for Chang et al. Summary effects were computed using inverse-variance weighted (IVW) method from each individual SNP. Dots represent the coefficient (B) and the extremities represent the 95% confidence interval of the odds ratio.

Using the 45 SNPs as IVs for PD, there was no link between genetically primed PD and the risk towards periodontitis (B = −0.0002, SE = 0.0002, *p* = 0.85) (Figure 3).

The MR-Egger analyses revealed no horizontal pleiotropy (periodontitis on risk of PD using the seventeen SNPs from the study of Teumer et al.: intercept 0.000, *p* = 0.32; periodontitis on PD using the eight from the study of SNPs Munz et al.: intercept 0.002, *p* > 0.05; and PD on risk of periodontitis: intercept 0.000, *p* = 0.53).

The Cochran’s Q statistic reported minor heterogeneity among the NPS. Leave-one-out meta-analyses reported proportionately within the included SNPs (Supplementary File S3).

The independency of the IVs for periodontitis and PD was assumed of confounding through a comprehensive search on GWAS catalogue (https://www.ebi.ac.uk/gwas/; accessed on 1 January 2021) [27].

## 4. Discussion

To the best of our knowledge, this is the first study employing a two-sample MR method to explore a causal association between PD and periodontitis in a bidirectional way, in European ancestry populations. Overall, our results do not support a bidirectional genetic liability between these two conditions. However, these results should be interpreted with caution given the scarcity of GWAS studies available so far on these two diseases.

Neurological conditions have been associated with periodontitis, such as Alzheimer’s Disease, dementia, or PD [5,9,28,29]. From a biological standpoint, the association of periodontitis with these intricate illnesses may be based on the influence of systemic inflammation and the systemic spreading of periodontal pathogens products with potential affection of brain tissues [30,31]. These processes might support the reported association of *Porphyromonas gingivalis* IgG levels with compromised delayed memory and calculation [32]. Remarkably, a preclinical study in mice showed that continuous oral application of *Porphyromonas gingivalis* caused neurodegeneration and formation of extracellular Aβ_42_ [33]. The potential impact of leukocytosis found in PD patients with periodontitis is still uncertain, as persistent elevated levels of white blood cells were linked to cardiovascular or diabetic related negative events [34,35,36].

This MR study presents a comprehensive analysis from large and fashionable datasets. Nevertheless, there are a number of potential limitations to discuss. MR computation is powerless to elucidate if the existence of one illness in a particular period of lifetime can influence the risk of developing another illness. In addition, when there is a strong genetic/environmental interaction present (as might be the case in periodontitis), MR may lead to an inaccurate conclusion. Furthermore, this analysis was based on European ancestry studies due to the scarcity of GWAS studies in other populations, therefore limiting the generalizability of these results.

The instruments used in periodontitis showed weak association with PD, since the *p*-value was higher than the ideal. In addition, the lack of overlapping instruments between the two genome studies of periodontitis may denote weak IVs. Furthermore, we highlight that the biological mechanisms on how these SNPs impact periodontitis are still imprecise.

The instruments for periodontitis presented weak association, as the *p*-values were 5 × 10^−6^ (lower than 5 × 10^−8^). Additionally, both GWAS studies did not present overlapping SNPs, which further denotes weak instruments for periodontitis. Likewise, of all SNPs included in the analysis, its biological relation with periodontitis and how they biologically act in this condition is still far from being understood.

Importantly, GWAS studies on periodontitis are prone to fail in the identification of consistent SNPs [18,19,20,37,38] because of the inconsistent definitions of periodontitis that are being used in different studies. Hence, studies shall employ up-to-date definitions of periodontitis following the American Academy of Periodontology/European Federation of Periodontology case definition [39], considering its variability [40].

Further MR studies using summary statistics from GWAS datasets of α synuclein and LRRK2 may explore the potential biological pathways between periodontitis and the risk towards PD.

## 5. Conclusions

In this bidirectional MR study, we found no convincing evidence supporting a bidirectional genetic liability between periodontitis and PD. Further GWAS research is warranted to explore the consistency of these results further.

## Figures and Tables

**Table 1:** Mendelian randomization results for the association between PD and periodontitis.

Method	Periodontitis on PD						Parkinson’s Disease on Periodontitis Using Chang et al., 2017 ^±^		
	Instrumental SNPs from Teumer et al. *			Instrumental SNPs from Munz et al. ^#^					
	B (SE)	*p*-Value	Q Statistic/*p* Value	B (Se)	*p*-Value	Q Statistic/*p* Value	B (Se)	*p*-Value	Q Statistic/*p* Value
IVW	−0.0003 (0.0003)	0.26	11.45/0.78	−0.0000 (0.0001)	0.99	4.30/0.75	−0.0001 (0.0001)	0.19	46.83/0.36
Weighten median	−0.0003 (0.0003)	0.37	-	0.0000 (0.0002)	0.93	-	−0.0001 (0.0001)	0.31	-
MR-Egger	−0.0008 (0.0006)	0.17	10.37/0.80	−0.0014 (0.0046)	0.76	4.19/0.65	−0.0002 (0.0002)	0.24	46.39/0.33
MR-RAPS	−0.0003 (0.0003)	0.27	-	−0.0000 (0.0001)	0.99	-	−0.0001 (0.0001)	0.31	-

IVW: inverse-variance weighted; MR: Mendelian randomization; RAPS: robust adjusted profile score; SNP: single-nucleotide polymorphism. Seventeen */Eight ^#^ SNPs served as periodontitis IVs. ^±^ Forty-five ± SNPs served as Parkinson’s disease IVs.

## Data Availability

Data will be provided upon reasonable request from the corresponding author.

## Supplementary Materials

The following are available online at https://www.mdpi.com/article/10.3390/genes12050772/s1, Supplementary File S1: Summary statistics for MR analysis of potential causal effect of periodontitis on Parkinson’s disease, Supplementary File S2: Summary statistics for MR analysis of potential causal effect of Parkinson’s disease on periodontitis, Supplementary File S3: Leave-one-out meta-analysis for (A) Munz et al., (B) Teumer et al. and (C) Chang et al.
