# Supplementary material for: Causal Association between Periodontitis and Parkinson’s Disease: A Bidirectional Mendelian Randomization Study
Source: Genes (Basel). 2021 May 19;12(5):772. doi: 10.3390/genes12050772 (PMC8159074; doi:10.3390/genes12050772)
Supplement: Supplementary file 1 [file genes-12-00772-s001.zip › Supplementary File S1.pdf]

Table S1. Summary statistics for Mendelian randomization analysis of potential causal effect of periodontitis on Parkinson's disease

SNP	Chr	Nearest genes/contig	Alleles: effect/other	Eaf†	Exposure: Periodontitis			Outcome: PD (37,688 cases and 1.4 million controls) [1]		
					Coefficient#	SE	*P* value	Coefficient#	SE	*P* value
Teumer *et al.* [2] (1,817 periodontitis cases vs. 2,215 controls)										
rs111571364	2	GPN1	T / G	0.02	1.24	0.2777	8.00E-06	0.0002	0.0015	0.89
rs12048046	1	ACTN2	T / C	0.90	0.42	0.0884	3.00E-06	0.0004	0.0006	0.50
rs12587630	14	FOXA1	A / G	0.98	0.99	0.2203	7.00E-06	-0.0013	0.0011	0.22
rs13237474	7	FAM180A	T / C	0.03	1.12	0.2152	2.00E-07	-0.0018	0.0011	0.10
rs1953021	9	C9orf150	T / G	0.71	0.30	0.0620	1.00E-06	-0.0004	0.0004	0.33
rs2009196	17	TIMP2	C / G	0.23	0.34	0.0723	2.00E-06	0.0001	0.0004	0.77
rs263063	19	JMJD2B	T / C	0.08	0.49	0.1073	6.00E-06	-0.0004	0.0006	0.51
rs28455997	8	CSMD1	T / C	0.84	0.39	0.0838	3.00E-06	0.0001	0.0005	0.83
rs28643277 ¥	10	ITGA8	A / C	0.35	0.28	0.0634	9.00E-06	-0.0001	0.0004	0.70
rs3128935	6	HLA-DOA	T / C	0.97	0.82	0.1847	9.00E-06	-0.0015	0.0010	0.15
rs6497031	15	RGMA	G / A	0.83	-0.61	0.1346	5.00E-06	0.0003	0.0005	0.56
rs6890783	5	ERGIC1	A / G	0.01	1.86	0.4093	5.00E-06	-0.0002	0.0016	0.89
rs72899866	2	LRP1B	A / T	0.22	0.33	0.0713	5.00E-06	0.0002	0.0004	0.58
rs73389468	10	C10orf91	C / T	0.98	1.02	0.2323	9.00E-06	0.0015	0.0014	0.29

rs7819988	8	SNTB1	T / C	0.55	0.28	0.0600	3.00E-06	0.0003	0.0004	0.37
rs78411303	3	PARP15	A / G	0.094	0.67	0.1415	3.00E-06	-0.0008	0.0007	0.27
rs78797168	6	IGF2R	A / G	0.93	0.56	0.1194	2.00E-06	0.0004	0.0007	0.52
Munz *et al.* [3] (4,924 periodontitis cases vs. 7,301 controls)										
rs11084095	19	SIGLEC5, AC018755.2	G / A	0.59	-1.17	0.030612	5,00E-08	-0.0003	0.00036	0.38
rs3780866	10	NUDT5	G / A	0.89	1.17	0.040816	8,00E-06	-0.0001	0.00042	0.83
rs6725179	21	RNU6-675P, TEX51	G / A	0.66	-1.15	0.035714	3,00E-06	-0.0003	0.00037	0.38
rs7031401	-	LINC01239, AL391117.1	A / G	0.92	-1.27	0.061224	6,00E-06	-0.0003	0.00065	0.60
rs729876	16	U91319.1	T / C	0.80	1.23	0.040816	1,00E-08	0.0000	0.00044	0.94
rs886431	12	AC004241.1	G / A	0.09	1.19	0.045918	4,00E-06	-0.0005	0.00047	0.26
rs9982623	21	MCM3AP	C / T	0.86	1.23	0.05102	9,00E-07	-0.0002	0.00053	0.70
rs9992954	4	MIR297, LYPLA1P2	C / T	0.84	-1.18	0.040816	9,00E-06	0.0005	0.00046	0.31

PD: Parkinson's disease; Chr: chromosome; Eaf: effect allele frequency; SE: standard error; SNP: single-nucleotide polymorphism
Seventeen/eight SNPs in two GWAS were used as instrumental variables for chronic periodontitis respectively.
¥rs28643277 (A / C) was used as proxy SNP (linkage disequilibrium R^2>0.8).

References

1. Nalls, M.A.; Blauwendraat, C.; Vallerga, C.L.; Heilbron, K.; Bandres-Ciga, S.; Chang, D.; Tan, M.; Kia, D.A.; Noyce, A.J.; Xue, A.; et al. Identification of novel risk loci, causal insights, and heritable risk for Parkinson's disease: a meta-analysis of genome-wide association studies. *Lancet Neurol.* 2019, *18*, 1091–1102.
2. Teumer, A.; Holtfreter, B.; Völker, U.; Petersmann, A.; Nauck, M.; Biffar, R.; Völzke, H.; Kroemer, H.K.; Meisel, P.; Homuth, G.; et al. Genome-

wide association study of chronic periodontitis in a general German population. *J. Clin. Periodontol.* **2013**, *40.* 977–985.
3. Munz, M.; Richter, G.M.; Loos, B.G.; Jepsen, S.; Divaris, K.; Offenbacher, S.; Teumer, A.; Holtfreter, B.; Kocher, T.; Bruckmann, C.; et al. Meta-analysis of genome-wide association studies of aggressive and chronic periodontitis identifies two novel risk loci. *Eur. J. Hum. Genet.* **2019**, *27*, 102–113.
