# Supplementary material for: Causal Association between Periodontitis and Parkinson’s Disease: A Bidirectional Mendelian Randomization Study
Source: Genes (Basel). 2021 May 19;12(5):772. doi: 10.3390/genes12050772 (PMC8159074; doi:10.3390/genes12050772)
Supplement: Supplementary file 1 [file genes-12-00772-s001.zip › Supplementary File S2.pdf]

Table S2. Summary statistics for MR analysis of potential causal effect of Parkinson’s disease on periodontitis

SNP	Chr	Nearest gene	Alleles: effect[*]/other	Eaf	Exposure: PD (20,184 cases and 397,324 controls)			Outcome: periodontitis (975 adult participants) [2]		
					Coefficient[#]	SE	*P* value	Coefficient[#]	SE	*P* value
rs10463554	5	PAM	T / C	NA	0,0629	0,0120	2,00E-07	0,0000	5.89e-05	0.43
rs10797576	1	SIPA1L2	T / C	0.135	0,1133	0,0137	8,00E-13	-0,0001	8.41e-05	0.09
rs10906923	10	FAM171A1	A / C	0.694	0,0726	0,0143	1,00E-08	0,0001	5.94e-05	0.22
rs11060180	12	OGFOD2	A / G	0.551	0,1054	0,0114	2,00E-20	0,0001	5.63e-05	0.36
rs11158026	14	GCH1	C / T	0.669	0,0943	0,0093	4,00E-16	-0,0001	5.87e-05	0.29
rs11343	16	COQ7	T / G	0.454	0,0677	0,0119	9,00E-11	-0,0001	5.702e-[illegible]	0.02
rs117896735	10	BAG3	A / G	0.015	0,5008	0,0569	2,00E-19	0,0001	0.0002	0.76
rs12456492	18	SYT4	G / A	0.315	0,0953	0,0117	6,00E-16	0,0000	5.99e-05	0.95
rs12497850	3	NCKIPSD	T / G	0.653	0,0726	0,0143	9,00E-09	0,0000	5.81e-05	0.39
rs12637471	3	MCCC1	G / A	0.802	0,1625	0,0151	2,00E-30	-0,0002	7.06e-05	0.03
rs13294100	9	SH3GL2	G / T	0.629	0,0834	0,0140	5,00E-13	0,0000	5.84e-05	0.89
rs14235	16	ZNF646	A / G	0.378	0,0770	0,0094	5,00E-12	0,0001	5.78e-05	0.08
rs1474055	2	STK39	T / C	0.126	0,1863	0,0191	6,00E-26	-0,0001	8.60e-05	0.41
rs17649553	17	ARHGAP27	C / T	0.779	0,2485	0,0139	1,00E-68	0,0000	6.64e-05	0.71
rs199347	7	KLHL7	A / G	0.588	0,0943	0,0093	4,00E-18	0,0000	5.65e-05	0.81
rs2280104	8	SORBS3	T / C	0.367	0,0677	0,0120	3,00E-08	0,0000	5.81e-05	0.46
rs2296887	10	GBF1	C / T	NA	0,0807	0,0142	2,00E-07	0,0001	7.92e-05	0.47
rs2414739	15	VPS13C	A / G	0.734	0,0943	0,0093	4,00E-14	0,0000	6.38e-05	0.68
rs2694528	5	ELOVL7	C / A	0.115	0,1398	0,0199	5,00E-15	-0,0001	0.0001	0.26

rs2740594	8	CTSB	A / G	0.753	0,0862	0,0117	6,00E-12	0,0000	6.33e-05	0.94
rs316619	15	LTK	C / T	NA	0,0534	0,0121	7,00E-06	0,0000	5.76e-05	0.4
rs329648	11	MIR4697	T / C	0.351	0,0862	0,0117	1,00E-13	0,0000	5.85e-05	0.54
rs34043159	2	IL1R2	C / T	0.352	0,0770	0,0094	5,00E-11	0,0000	5.85e-05	0.69
rs34311866	4	TMEM175	C / T	0.184	0,2070	0,0145	1,00E-50	-0,0001	7.30e-05	0.05
rs356182	4	SNCA	G / A	0.349	0,2852	0,0115	5,00E-122	-0,0001	5.95e-05	0.29
rs35749011	1	GBA	A / G	0.012	0,5447	0,0441	3,00E-35	0,0002	0.0002	0.36
rs3793947	11	DLG2	G / A	0.558	0,0726	0,0119	4,00E-09	0,0001	5.66e-05	0.35
rs4073221	3	SATB1	G / T	0.132	0,0953	0,0163	2,00E-08	0,0001	8.41e-05	0.25
rs4653767	1	ITPKB	T / C	0.685	0,0834	0,0118	2,00E-11	-0,0001	6.19-05	0.27
rs4784227	16	TOX3	T / C	0.265	0,0862	0,0140	1,00E-10	-0,0001	6.49e-05	0.44
rs591323	8	MICU3	G / A	0.726	0,0943	0,0140	2,00E-11	0,0000	6.17e-05	0.57
rs62120679	19	LSM7	T / C	0.31	0,0770	0,0142	7,00E-07	0,0000	6.14e-05	0.49
rs6416935	17	MED13	G / T	NA	0,0780	0,0142	4,00E-07	0,0001	7.69e-05	0.38
rs6430538	2	TMEM163	C / T	0.55	0,1165	0,0113	8,00E-24	0,0001	5.69e-05	0.34
rs67460515	3	SPTSSB	C / T	NA	0,0573	0,0096	1,00E-06	-0,0001	6.01e-05	0.19
rs6812193	4	FAM47E	C / T	0.63	0,0834	0,0118	1,00E-14	-0,0001	5.74e-05	0.36
rs76904798	12	LRRK2	T / C	0.137	0,1398	0,0155	1,00E-19	0,0001	7.90e-05	0.24
rs78738012	4	ANK2	C / T	0.106	0,1222	0,0181	5,00E-11	0,0000	9.28e-05	0.66
rs8005172	14	GALC	T / C	0.424	0,0770	0,0119	9,00E-11	-0,0001	5.62e-05	0.19
rs8118008 ¥	20	DDRGK1	A / G	0.609	0,0677	0,0120	2,00E-06	0,0000	5.72e-05	0.64
rs9275326	6	HLA-DRB6	C / T	0.895	0,1625	0,0218	1,00E-13	0,0000	9.19e-05	0.88

rs943437	6	CCN6	A / G	NA	0,0899	0,0163	6,00E-08	-0,0001	8.42e-05	0.39
rs9468199	5	ZNF184	A / G	0.172	0,1044	0,0138	1,00E-12	0,0000	7.37e-05	0.71
rs9516970	13	-	A / G	NA	0,0630	0,0120	5,00E-07	-0,0001	5.93e-05	0.07
rs9568188	13	CAB39L	T / C	NA	0,0661	0,0120	4,00E-07	0,0000	6.27e-05	0.61

PD: Parkinson's disease; Chr: chromosome; Eaf: effect allele frequency; SE: standard error; SNP: single-nucleotide polymorphism

*Effect allele carrier has increased risk of PD; #Coefficient: B estimate

These 45 SNPs were used as instrumental variables for PD.

[¥] rs8118008 (A/G) was used as proxy SNP (linkage disequilibrium $R^2>0.8$).

References

1. Chang, D.; Nalls, M.A.; Hallgrímsdóttir, I.B.; Hunkapiller, J.; Brug, M. van der; Cai, F.; Kerchner, G.A.; Ayalon, G.; Bingol, B.; Sheng, M.; et al. A meta-analysis of genome-wide association studies identifies 17 new Parkinson's disease risk loci. *Nat. Genet.* **2017**, *49*, 1511–1516.
2. Offenbacher, S.; Divaris, K.; Barros, S.P.; Moss, K.L.; Marchesan, J.T.; Morelli, T.; Zhang, S.; Kim, S.; Sun, L.; Beck, J.D.; et al. Genome-wide association study of biologically informed periodontal complex traits offers novel insights into the genetic basis of periodontal disease. *Hum. Mol. Genet.* **2016**, *25*, 2113–2129.
