# Supplementary figures and images for: Causal Association between Periodontitis and Parkinson’s Disease: A Bidirectional Mendelian Randomization Study
Source: Genes (Basel). 2021 May 19;12(5):772. doi: 10.3390/genes12050772 (PMC8159074; doi:10.3390/genes12050772)

Supplementary File S3. Leave-one-out meta-analysis for (A) Munz et al., (B) Teumer et al. and (C) Chang et al.

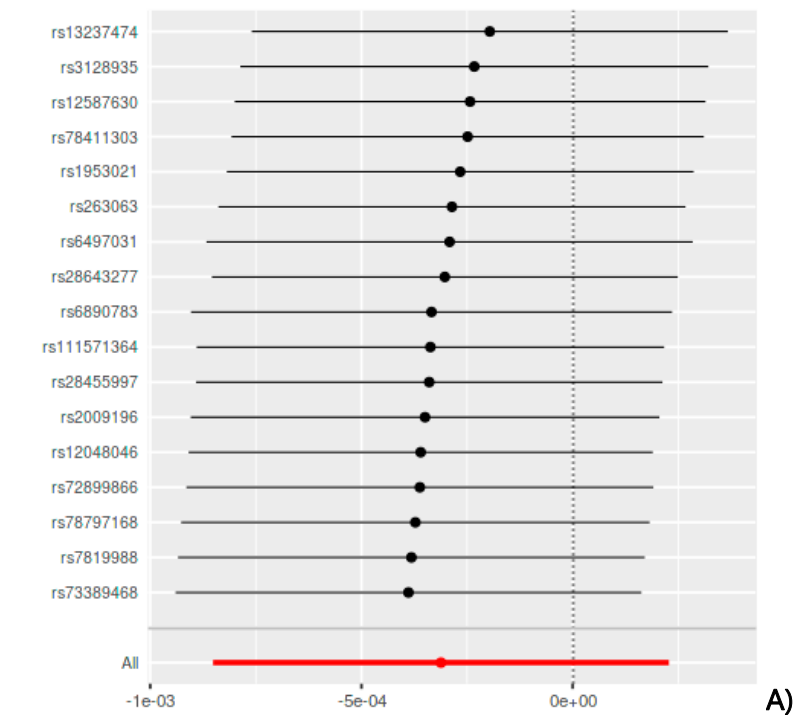

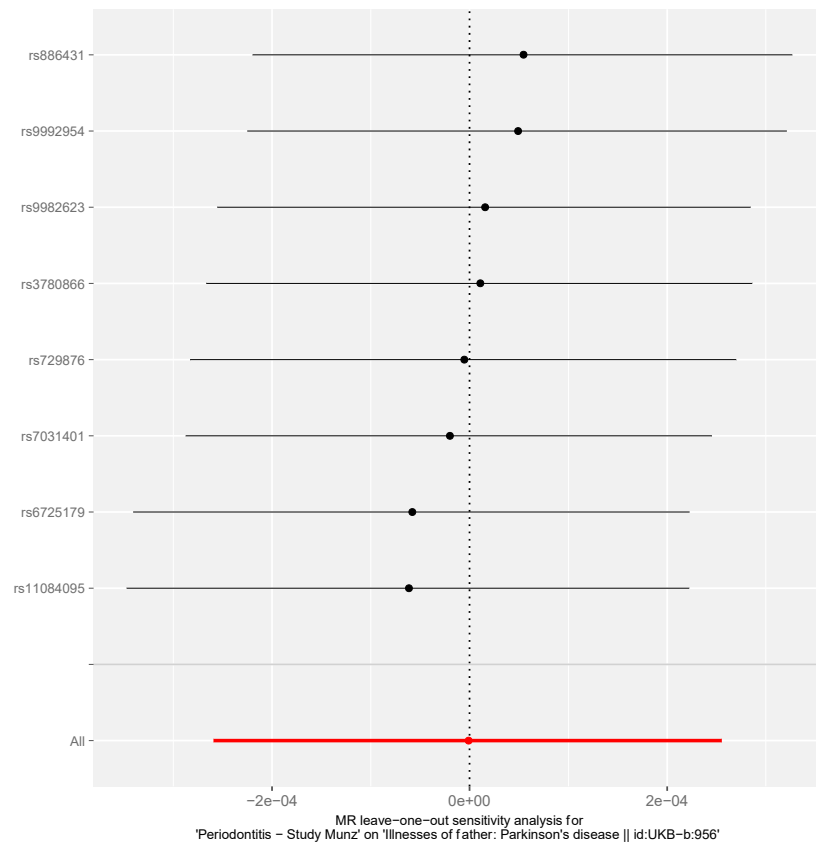

B)

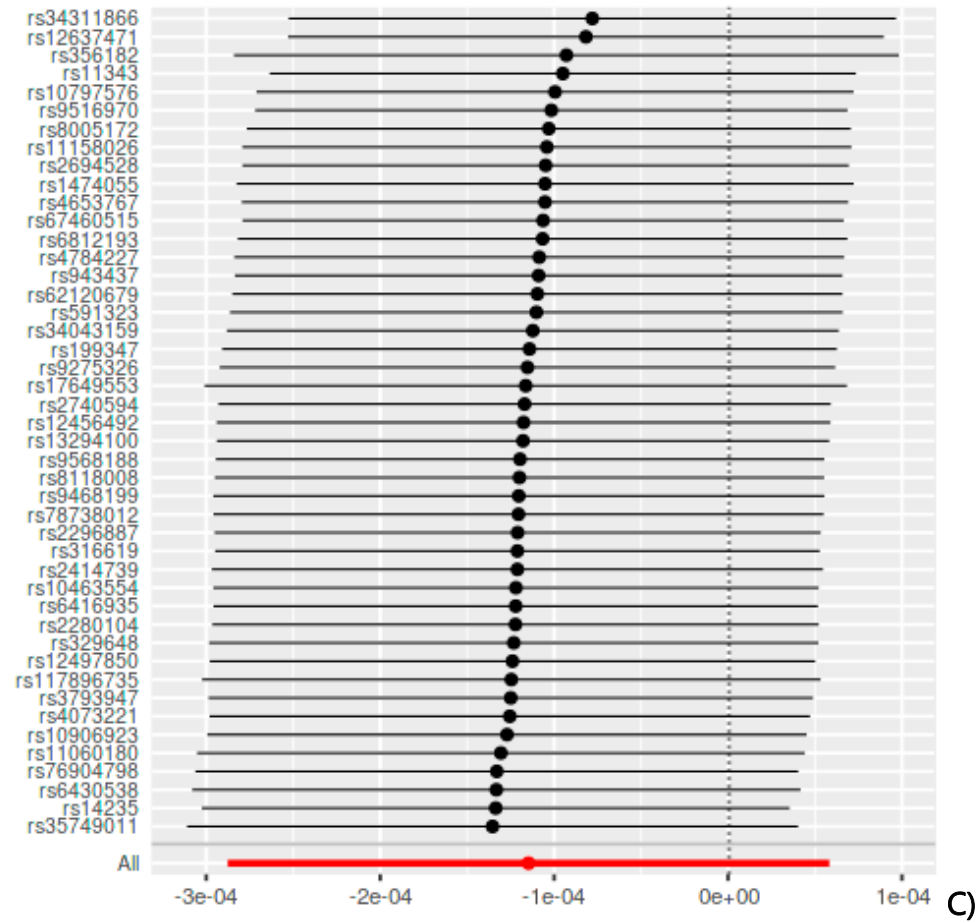

Supplement: Supplementary file 1 [file genes-12-00772-s001.zip › Supplementary File S3.pdf]
